# Assessment of clinical competencies using clinical images and videos “CIVA”

**DOI:** 10.1186/1472-6920-13-78

**Published:** 2013-05-30

**Authors:** Nabil D Sulaiman, Hossam Hamdy

**Affiliations:** 1 Department of Family and Community Medicine and Behavioural Sciences, University of Sharjah, Sharjah, United Arab Emirates; 2 College of Medicine, University of Sharjah, Sharjah, United Arab Emirates

## Abstract

**Background:**

This paper describes an assessment approach of clinical competencies which widens the number of problems and tasks evaluated using videos and images.

**Method:**

Clinical Image and Video Assessment (CIVA) was used to assess clinical reasoning and decision making of final year medical students. Forty to fifty clinical videos and images supported by rich text vignette and reviewed by subject matter experts were selected based on examination blueprints for analysis. CIVA scores were correlated with OSCE, Direct Observation Clinical Encounter Exam (DOCEE) and written exam scores, using the 2-sided Pearson correlation analysis, and their reliability was analyzed using Cronbach’s Alpha Coefficient. Furthermore, students personally evaluated the CIVA using a 5- point Likert scale.

**Results:**

CIVA and OSCE scores showed a high correlation (r = 0.83) in contrast with the correlation scores of the written examination (r = .36) and the DOCEE (r = 0.35). Cronbach’s Alpha for the OSCE and CIVA for the first batch was 0.71 and 0.78. As for the second batch it was 0.91 and 0.91 respectively. Eighty-two percent of students were very satisfied or satisfied with the CIVA process, contents and quality.

**Conclusions:**

A well constructed CIVA type assessment with a rich authentic vignette and good quality videos and images could be used to assess clinical reasoning and decision making of final year medical students. CIVA is an assessment tool which correlates well with OSCE, compliments the written and DOCEE and is easier to conduct at a possibly reduced cost.

## Background

Clinical competency is an outcome which entails several skills directly related to the holistic approach to patient care. Clinical reasoning, as a construct, guides hypothesis driven history taking, physical examination, diagnosis and management. Assessing clinical reasoning as a reflection of medical students’ ability in patient care is key to medical education. Researchers have shown that physicians use both analytical and non-analytical “pattern recognition” approaches, alternating between both in varying degrees [1]. As a result, developing, teaching and assessing clinical reasoning has always been a challenge in medical education [2-4].

Multiple tools and instruments have been described and used in assessment of clinical competencies, consequently Van der Vleuten [5] used Millers Pyramid to hierarchically place them in accordance to their assessment outcomes, those being - Knows, Knows how, Shows and Does. Moving upwards towards the “does” level increases the assessment of the instrument’s authenticity, bringing it closer to reality and the workplace. The balance between instrument validity, authenticity, reliability, practicality, educational impact and cost is what all assessment systems aim to achieve [6], in order to have an appropriate impact on student learning [7].

The most ideal way to judge students’ or physicians’ clinical competencies and clinical reasoning, would be through direct observation of a large number of real patient encounters in the normal work place environment. This is usually difficult to achieve because of the lack of suitable patients and patient safety regulations. All commonly described clinical assessment instruments – ‘A’ type Multiple Choice Questions (MCQs), Extending Matching Questions (EMQs), Key Feature Examinations, Objective Structured Clinical Examinations (OSCEs), Mini C-Ex [8], Direct Observation Clinical Encounter Examination (DOCEE) [9] have their limitations. Most assessment systems use these instruments in combination in order to achieve the criteria set by Van Der Vleuten, as mentioned earlier. The College of Medicine at the University of Sharjah, follows an integrated, outcome based curriculum, that is conveyed to the student in a problem based learning (PBL) manner. Clinical skills are introduced in Year One and constitute one of the several crucial vertical themes within the curriculum.

In assessing students’ clinical competencies, test blue prints, written exams, OSCEs and DOCEEs are used during the clerkship phase and exit examinations to assess a medical student’s knowledge, skills and attitudes. The OSCEs, particularly in the clerkship phase comprise around 15 interactive “7 minute” stations using simulated patients in contrast to the DOCEEs, where the medical student on average, examines four real patients. The DOCEE is designed to observe and assess the student’s full encounter with real patients. Each DOCEE station spans over a period of 30 minutes where the examiner goes through a standardized check list to assess the student’s performance. As for the student’s depth of clinical knowledge, it is tested using 100 A-type Multiple Choice Questions (MCQs), in addition to 100 Extended Matching Questions (EMQs). All final exit exams are conducted over a period of one week.

In order to increase the validity and reliability of assessment of clinical competencies and ensure wider sampling from the broad world of clinical context, we have introduced an additional instrument known as “Clinical Images and Video Assessment” (CIVA). It’s rationale is based on assessing students’ pattern recognition competencies in generating diagnostic hypotheses and decision making in patient management.

CIVA is a computer-projected, class administered test, comprising 40–50 scenario rich electronic stations (slides) that demonstrate clinical images or videos, followed by a number of short questions that relate to clinical reasoning and decision making regarding the patient that had been described in the scenario. By increasing the number of problems and practice context, the number of clinical tasks tested increases.

The aim of this study is to evaluate the validity, reliability and the students’ perception of CIVA as an additional examination tool used for the evaluation of clinical reasoning and decision making.

### Context

The College of Medicine, established in 2004, adopted an outcome -based curriculum, with its main approach to teaching being through Problem-based Learning (PBL). The pre-clerkship phase starts in the foundation year, following which students during the years after, those being year 1 and 2 are subjected to clinical work that is accompanied by community training in year 3 and continued through in years 4 and 5. In addition to the acquisition of knowledge, the curriculum focuses on introducing clinical skills training as early as year one.

Starting from Year 2, in addition to OSCEs, CIVAs are also included to assess clinical reasoning including pattern recognition of clinical signs, decision making as well as medical writing skills, such as prescriptions and referral letters. To ensure content validity, an assessment blueprint is designed beforehand based on outcome objectives, problems and clinical tasks (Table 1) [10].

**Table 1:** Final MBBS exam

**Organ system**	**Problem**	**Practice context**		**Age**			**Clinical task**					**Test instrument**	
		**1**	**2**	**1**	**2**	**3**	**1**	**2**	**3**	**4**	**5**	**6**	
Musculoskeleta & Rheumatology	Fracture humerus	**x**			**x**				**x**	**x**			CIVA
	Dislocation shoulder	**x**			**x**				**x**	**x**	**x**		CIVA
	Rheumatoid arthritis		**x**			**x**		**x**		**x**			CIVA/OSCE
Peripheral Nervous (PNS)	Weakness LL		**x**		**x**	**x**		**x**					OSCE
Cardiovascular	Coronary angiography		**x**		**x**				**x**				CIVA
	Atrial flutter	**x**			**x**			**x**	**x**	**x**			CIVA
	Post MI advice		**x**			**x**					**x**	**x**	OSCE
	Postural hypotension		**x**			**x**		**x**	**x**				OSCE
Respiratory	Horner syndrome		**x**			**x**		**x**	**x**				CIVA
	Stridor	**x**		**x**				**x**					CIVA
	Resp distress in new born	**x**		**x**				**x**					CIVA
	Pneumothorax	**x**				**x**		**x**	**x**	**x**			OSCE
	Recurrent cough		**x**	**x**			**x**	**x**	**x**	**x**	**x**		0SCE
Gastrointestinal	Shifting dullness		**x**		**x**			**x**					CIVA
	Acute abdomen		**x**			**x**		**x**			**x**		OSCE
	Per rectal bleeding							**X(PR)**		**x**			OSCE
GenitoUrinary / Renal	Catheterization	**x**			**x**	**x**		**x**	**x**				CIVA/OSCE
	Inguinal hernia		**x**			**x**					**X(consent)**		OSCE
Endocrine	Cushing’s syndrome		**x**		**x**			**x**	**x**	**x**			CIVA
	Developmental milestones		**x**	**x**				**x**	**x**				CIVA
Reproductive	PCOS		**x**		**x**			**x**	**x**				CIVA
	Pap smear		**x**		**x**			**x**		**x**			CIVA
	Episiotomy	**x**			**x**			**x**	**x**				CIVA
	Irregular bleeding		**x**			**x**	**x**		**x**	**x**			OSCE
Central Nervous (CNS)	Heel shin test		**x**		**x**			**x**	**x**				CIVA
	Intention tremor		**x**		**x**					**x**	**x**		CIVA
	Lumbar puncture		**x**		**x**			**x**	**x**				CIVA
	Babinski sign		**x**		**x**			**x**	**x**				CIVA
	Febrile convulsion	**x**		**x**				**x**	**x**		**x**		CIVA
Hematopoietic (Hem & Lymphatics)	Hemarthrosis		**x**		**x**			**x**	**x**				CIVA
	Iron deficiency anemia		**x**	**x**				**x**	**x**				CIVA
	Cervical LN pathy		**x**		**x**			**x**	**x**				CIVA
Integumentary (Skin & related) + Connective Tissue	Purpura		**x**		**x**			**x**	**x**				CIVA
	Burns	**x**		**x**							**x**	**x**	CIVA
	Herpes zoster		**x**		**x**			**x**			**x**		CIVA
Special Senses	Hearing tests		**x**	**x**				**x**	**x**				CIVA

Blueprint University of Sharjah, College of Medicine / Clinical Skills Program for CIVA and OSCE only.

**Practice Context**: 1 emergency, 2 non emergency. **Age**: 1 child, 2 Adult, 3 Elderly. **Clinical tasks**: 1 History, 2 Physical Examination, 3 Diagnosis, 4 Investigation, 5 Treatment, 6 Follow-up. **Test instrument**: OSCE -Objective Structured Clinical Exam, CIVA - Clinical Image & Video Assessment.

The Clinical Skills team (CST) is comprised of six clinical tutors; a lecturer, a senior faculty (NS) as well as clinicians from the University of Sharjah Hospital. The team is responsible for the assessment of clinical skills. The team director is a member of the assessment committee, which is responsible for formulating each exam’s master blueprint and monitoring its quality. The CST is responsible for developing and implementing OSCEs and CIVAs in accordance with the master test blueprint, which includes all domains assessed by different assessment tools, written A-type, Extended Matching, OSCE, CIVA and DOCEE. Each OSCE and CIVA is reviewed by the assessment committee for validity and mapping based on the master exam blueprint. The CST has developed a large database of clinical videos and images which are revised frequently for face and content validity. Videos and images are selected to assess outcomes of clinical reasoning and decision making (diagnosis, management, follow-up). The videos/images are supplemented with a rich but short clinical vignette which is revised for authenticity by subject matter experts (consultant clinicians). In the final exit examination at the end of the clerkship phase, CIVA is comprised of 50 stations (Table 2). For each image, the questions as well as the model answers are reviewed meticulously to prevent overlap. The CIVA and OSCE are offered on the same examination day. Students are divided into two groups which alternate in taking the OSCE and CIVA. A few days prior to the test, the CIVA is pilot tested to ensure good quality of images and sounds and to estimate the time required. The CIVA is offered to a group of 25–30 students. Students write their answers in a structured answer books, which are then marked by members of the CST according to the model answers and cross checked by second assessors. The weight of each station differs according to the number of questions related to that station. The time required for preparing the CIVA station varied according to the complexity of the station. Once developed and verified, the CIVA is saved in a CIVA bank for future use. The average time for displaying/answering each station is two minutes and the time needed for marking ranges between 30 to 60 seconds.

**Table 2:** **Examples of CIVA stations**, **University of Sharjah, College of Medicine / Clinical Skills Program**

**Example 1**	**Example 2**
The video shows a patient demonstrating a raised Jugular Venous Pressure (JVP)	The Video features a patient having a major seizure
This 67-year-old man featured in this video complains of severe dyspnoea at rest. He states that he suffered a heart attack one year ago. His pulse = 130 bpm, Respiratory rate = 34, BP = 130/85 mmHg. On examination of the neck the test in the video was performed:	A 33-year-old lady is known to frequently experience the event shown in the following video. She is otherwise healthy, doesn’t use illicit drugs & is doing well at her job.
a. What is the finding?	a. Describe the event seen.
b. What is your diagnosis?	b. Name 3 most likely underlying causes in this patient.
c. Write the most appropriate prescription for this patient based on the history and your diagnosis.	c. State the first aid management.

## Methods

Two final year students batches (n = 52 and n = 95) sitting for the final exit examination were studied. Students' perception of the CIVA was obtained though a questionnaire. Quantitative responses using a 5-point Likert scale (strongly disagree, agree, undecided, agree and strongly agree) were recorded. The question items were related to the clinical problem, quality of the image and video and time allowed for each station. The overall response rate was 72%.

Qualitative data was collected from the free response of the students and analyzed to identify common emerging themes.

### Statistical analysis

Students’ final scores, for the two batches, in CIVA, OSCE, DOCEE and MCQ were subsequently compiled in one file for further analysis. Pearson Correlation Coefficient (r) was used to measure the Correlation of CIVA with the OSCE, DOCEE and MCQ. The level of significance was set at 5%. The Cronbach’s Alpha Coefficient was calculated as a measure of exam reliability. Because question items in the CIVA and OSCE exams differed in number and scoring among the two batches, a separate reliability measurement was conducted for each.

## Results

**Figure 1:**
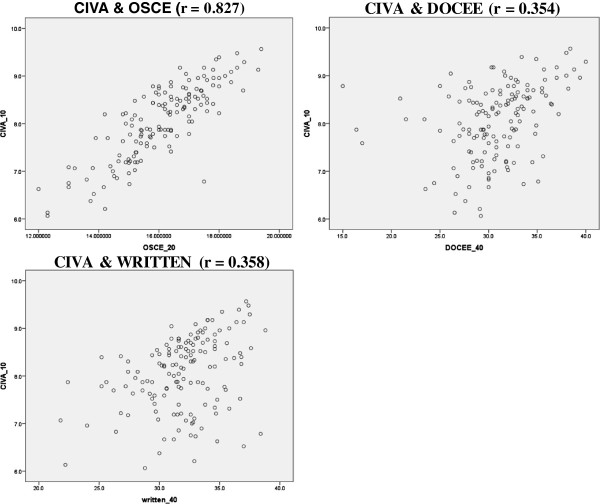
Scatter plots for correlations showing Pearson’s correlation (r).

Table 3 and Figure 1 show the correlation (r) between CIVA and OSCE, the DOCEE and written exams grades. The strongest correlation was found to be between CIVA and OSCE (r = 0.83, p < 0.001). However, CIVA grades correlate less with written forms (r = 0.36, p < 0.001) and DOCEE grades (r = 0.35, p < 0.001). Cronbach’s Alpha for the OSCE and CIVA for the first batch of students was 0.71 and 0.78 and for the second batch was 0.91 and 0.91 respectively, indicating good reliability.

**Table 3:** Correlations coefficient (r) between CIVA and other modes of assessment (N of students = 147)

	**Written**	**DOCEE**	**OSCE**	**CIVA**
Written	1	.40^**^	.42^**^	.36^**^
DOCEE	.40^**^	1	.461^**^	.35^**^
OSCE	.42^**^	.46^**^	1	.83^**^
CIVA	.36^**^	.35^**^	.83^**^	1

**. Correlation is significant at the 0.01 level (2-tailed).

Qualitative feedback following CIVA from the majority of the students was positive for both educational and technical quality. The majority of students agreed/strongly agreed that: the overall knowledge tested was fair (86%), was relevant and correlated well to the curriculum (89%) and reflected common case scenarios (84%). Technically CIVA ran smoothly (93%), sound and video effects were very good (96%) and time allocated was fair (80%). Suggested areas for improvement were *“time allocated was too long, I could have done it in 30 minutes*”, “*few stations have a lot of time, others is no time, but it was good overall*”, *“ better quality of pictures”, “too many stations I felt sleepy”, “some x-rays were not clear”, “one slide for the eye was not obvious”.*

## Discussion

The assessment method of Clinical Images and Videos Assessment (CIVA) at the University of Sharjah can be considered as a valid and reliable additional method in the assessment toolbox. It provides an opportunity to test a large sample of clinical skills in a short time with little cost and resources. The specific skills of clinical diagnosis for CIVA include pattern recognition of signs, interpretation and decision making as well as medical writing skills such as referral letters and prescription writing. The topics chosen were based upon the defined curriculum outcome competencies, in line with GMC learning outcomes [11] and the assessment of these outcomes [7], uses the principles of the Millers Pyramid [12] in moving assessments higher up the hierarchical scale towards the realism of the clinical situation [5]. This model is so far useful with regard to assessment using different assessment instruments that are appropriate at each level of the pyramid. The CIVA like the OSCE is more appropriate to the second and third level of the pyramid.

CIVAs and OSCEs assess similar constructs of clinical reasoning and decision making, but the value of CIVA is the increased size and number of tasks in different simulation contexts and presentations such as “emergencies” which are difficult and more expensive to simulate in OSCEs, such as seizures, severe asthma, acute cardiac conditions and trauma. However, CIVA is not an alternative tool to OSCE, as it is not ideal for assessing communication skills, history taking, physical examination or procedural skills. The main advantage of CIVA is the standardized mean of assessing a large number of students in a short time.

The high correlation with OSCE (r = 0.83, p <0.001) indicates similarity of constructs assessed including clinical reasoning and decision making. It could also be due to a priming effect because both tests were administered on the same day. However, the low correlations with written (r = 0.36, p <0.001) and DOCEE grades (r = 0.35, p <0.001) reflect the differences of the constructs measured. The written exams are an effective mean of assessing knowledge and other domains, and the DOCEE evaluates the holistic approach to patient’s care.

Although CIVA is cheaper to administer, it does require significant time for getting it right before adding the station on the database and for marking the answer booklets.

## Conclusion

CIVA provides an excellent reliable and valid tool to be added to the assessment tool box in order to enhance the overall assessment of how competent medical students are at various phases of the curriculum. It assesses a large number of clinical signs based on real patients. When well designed and enhanced with rich text and a real patient’s scenario, it can replace several OSCE stations and overcome some logistical issues encountered during OSCEs and DOCEEs. It is cheaper and requires much less personnel than the OSCE. However, it needs significant time to review in order to improve content validity and to mark the answer books.

## Competing interests

The authors declare that they have no competing interests.

## Authors’ contributions

NS conceived of the study and participated in its design and coordination as well as interpretation of results and helped to draft the manuscript. HH participated in the study design, interpretation of results and helped to draft and revise the manuscript critically. Both authors read and approved the final manuscript.

## Pre-publication history

The pre-publication history for this paper can be accessed here:

http://www.biomedcentral.com/1472-6920/13/78/prepub
